# MicroRNAs in HIV-1 infection: an integration of viral and cellular interaction at the genomic level

**DOI:** 10.3389/fmicb.2012.00306

**Published:** 2012-08-24

**Authors:** Neil H. Tan Gana, Tomohiro Onuki, Ann Florence B. Victoriano, Takashi Okamoto

**Affiliations:** Department of Molecular and Cell Biology, Nagoya City University Graduate School of Medical SciencesNagoya, Japan

**Keywords:** microRNA, HIV-1 mechanisms, transcription factors, targets

## Abstract

The microRNA pathways govern complex interactions of the host and virus at the transcripts level that regulate cellular responses, viral replication and viral pathogenesis. As a group of single-stranded short non-coding ribonucleotides (ncRNAs), the microRNAs complement their messenger RNA (mRNA) targets to effect post-transcriptional or translational gene silencing. Previous studies showed the ability of human immunodeficiency virus 1 (HIV-1) to encode microRNAs which modify cellular defence mechanisms thus creating an environment favorable for viral invasion and replication. In corollary, cellular microRNAs were linked to the alteration of HIV-1 infection at different stages of replication and latency. As evidences further establish the regulatory involvement of both cellular and viral microRNA in HIV-1-host interactions, there is a necessity to organize this information. This paper would present current and emerging knowledge on these multi-dimensional interactions that may facilitate the design of microRNAs as effective antiretroviral reagents.

## Introduction

The human immunodeficiency virus 1 (HIV-1) is the retroviral agent causing acquired immunodeficiency syndrome (AIDS), a disease leading to systemic failure of the immune system with life threatening consequences. The decades old magnanimous problem of HIV-1 infection has challenged researchers to address its control and eradication. One of the most recent strategies introduced is the use of small non-coding ribonucleotides (ncRNAs) which includes microRNAs (Arbuthnot, 2011). The microRNAs are ubiquitous ~22–25 nt endogenously expressed ncRNAs targeting specific messenger RNA (mRNA) sequences, thus inducing its degradation or effecting translational inhibition. As proven vital regulatory components of viral infection and immunity (Huang et al., 2011), microRNAs can be directed to target viral and cellular transcripts to suppress infection. In fact, numerous studies have been proposed to integrate cellular microRNAs as nucleotide-based therapy for HIV (Boden et al., 2004; Lo et al., 2007; Aagaard et al., 2008). However, HIV-1 is a fastidious mutant consequently making cellular microRNAs prone to losing its viral transcript target efficacy as constant genome revisions occur in the course of viral evolution. Thus, simultaneous expression of microRNAs aimed to repress multiple HIV-1 targets may deter the effects of escape mutants. In another scenario, the cellular microRNAs can target host gene products that regulate cell defense responses. Once the issues of microRNA off-target effects, cell toxicity and delivery systems are addressed, the development of microRNAs as an anti-HIV-1 therapeutic strategy becomes more realistic (Boden et al., 2007; Liu et al., 2011). Now, the greater challenge is to determine the specific roles of the current inventory of 1921 human and three HIV-1 microRNAs (Kozomara and Griffiths-Jones, 2011) in HIV-1 infection. This difficult task of functional assignments correlated to microRNA-mRNA interactions has been made easier with genomics-based predictive tools in the recent years (Tan Gana et al., 2012). In addition, significant improvements on techniques for microRNA discovery and functional elucidation are likely to further expand these emerging interactive networks.

**Figure 1:**
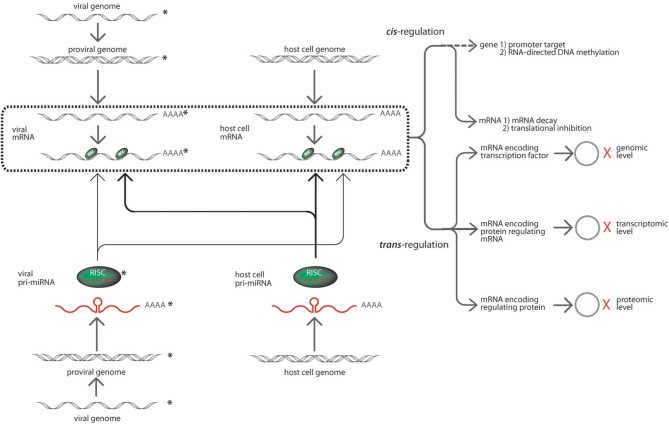
**The prospective targeting interactions of HIV-1 and cellular microRNAs (modified from Cullen, 2006).** The interactions among HIV-1 and cellular microRNAs with their corresponding targets may occur in several modules. For instance, HIV-1 encoded microRNAs processed via the host RNAi machinery and incorporated into RISC (in green with the mature microRNA in red) are sourced from HIV-1 pro-viral strands (grey double helix) initially from precursor microRNAs (red lines with poly adenines). These HIV-1 microRNAs can target its viral transcripts or the cellular transcripts. The targeting interactions of microRNAs are shown in solid light arrow lines. In corollary, cellular microRNAs derived from precursor microRNAs (red lines with poly adenines) generated by the host cell genome (grey double helix). The host cellular microRNAs are encoded in the same manner and can target both viral and cellular transcripts where the targeting interactions are shown in solid bold arrow lines. The targeting of mRNA transcripts happens in a highly specific Watson and Crick base-pairing with either complete complementation or seed region complementarity. The box in bold broken lines consolidates all targeting events of the various microRNA-initiated regulatory activities within the systems biology of host-virus interaction. The type of microRNA silencing mechanisms may be grouped as a *cis*- and *trans*-regulation event. The *cis*-regulation event involves microRNA targeting of mRNAs initiating post-transcriptional regulatory responses via mRNA degradation and translational inhibition. Whereas *trans*-regulation is a tripartite regulatory event which include expression variation of microRNA target genes regulating various viral and cellular activities such as transcription factors, RNA regulatory proteins, interactive genes. The cascades of events cause changes in viral and cellular activities inducing transcriptional regulation, transcriptional variation and protein translational modifications as indicated by the hollow circles = protein products; X (in red) = regulation of expression. The HIV-1 components are distinguished from host cell components with asterisks beside the drawings.

Whereas the current knowledge on cellular and viral microRNA functions involved in HIV-1 infection is still considerably few, consensus evidences suggest complex interactions (Chiang and Rice, 2011; Sanchez-Del Cojo et al., 2011). Figure 1 implies that microRNA regulation is anchored on genomic information processing on four scenarios that may possibly explain the confounded nature of their effects in virus-infected host systems. First, HIV-1 infection alters host microRNA networks to initiate successful viral invasion and latency, thus, affecting global host microRNA regulome. Second, HIV-1 microRNAs are produced from both sense and antisense transcripts to target either its own viral transcript or host genes for immune compromise. Third, the host microRNA systems may consequentially target the HIV-1 genomic elements or its genes to innate immune responses. Fourth, the interplay of microRNA and target mRNA between host and HIV-1 can be organized into regulatory modules (*cis*- and *trans*- regulation) of essential biochemical pathways as critical determinants of host cell fate and survival. This framework would be the basis of our paper discussion covering an update on the current information on microRNA biogenesis and mechanisms involved in host-virus interactions. Also the paper would contain recently elucidated cellular and viral microRNA functions in HIV-1 infection from computational and experimental literature. Lastly, the integration of information would define future roles of microRNAs in HIV-1 control.

## MicroRNA biogenesis pathways and mechanisms

**Figure 2:**
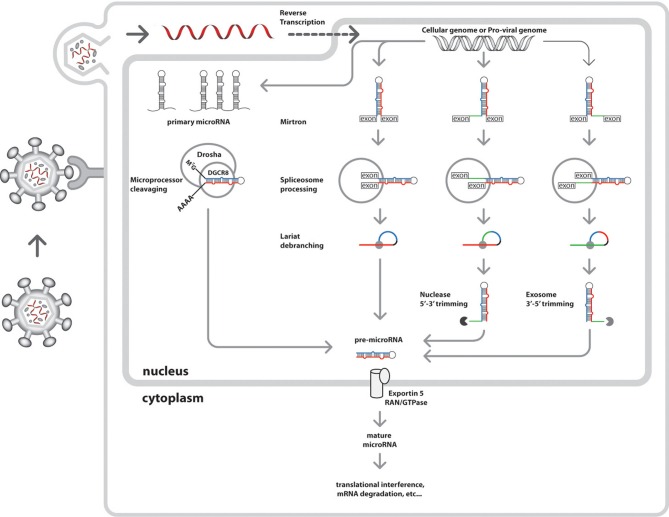
**Nuclear events of the integrated cellular and HIV-1 microRNA biogenic pathways.** The nucleus of the host cell is the central site of both cellular and viral microRNA biogenesis. Initially, HIV-1 virion particles attach to host cells via CD4 receptors signaling viral attack. This would be followed by HIV-1 particle fusion with the cell membrane and uncoating to load its RNA genome into the cytoplasm. The viral replicase enzyme facilitates production of more RNA genome later to be shuttled into the nucleus for viral transcription and integration into cellular genome. HIV-1 microRNA biogenesis is synchronous to cellular microRNA production in the host cell nucleus where other microRNA biogenic enzymes are present. Independent nuclear events of miRNA biogenesis have several modes of pre-microRNA generation (viral and cellular), initially from primary microRNA transcribed by either RNA polymerase II or III. he canonical pathway is undertaken by intergenic microRNAs resulting from microprocessor cleavage (Drosha and DGCR8) of pri-microRNA transcripts into pre-microRNAs, An alternative pathway for intron-coded microRNAs called mirtrons produce pre-microRNAs via splicing by spliceosomes and debranching by lariat debranching enzyme (Ldbr). There are three possible variants of mirtron processing, namely regular, the 5′ tailed mirtrons (subject to nuclease processing) and 3′ tailed mirtons (subject to exosome processing) (Westholm and Lai, 2011; Westholm et al., 2012). The sections of these pre-microRNA variants are shown in different colors, which the future main mature microRNAs are in **blue**, the secondary mature microRNAs are in **red**, the loops in black, and the branches are in **green**. Once generated, the pre-miRNAs are ready cytoplasmic shuttling, where further processing into mature microRNAs are achieved. Lately, a new biogenic pathway has been proposed for a set of splicing-independent mirtrons called simtrons which independent from DGCR8, Dicer, Exportin-5, or Argonaute 2 (Ago2) (Havens et al., 2012) (*not shown*).

The genomic locations of the microRNA gene progenitors of the ~100 nt, 5′ methyl-7G capped and 3′ poly-adenylated primary-microRNAs (pri-microRNA) transcribed by either RNA II or III polymerase determine the mode of microRNA biogenesis (Figure 2). The canonical pathway utilizes the microprocessor complex, an interaction between Drosha RNase III enzyme (Faller and Guo, 2008) and DiGeorge critical region gene 8 (DGCR8) (Faller et al., 2010), a ribonuclease binding protein (RBP) to cleave the pri-microRNA into 70 nt preliminary-microRNAs (pre-microRNAs). While, a non-canonical pathway is followed by mirtrons, a group of intron-derived pre-microRNAs utilizing spliceosomes (Okamura et al., 2007, 2008; Berezikov et al., 2010). Recently, an emerging mode of biogenic pathway has been proposed for a set of splicing-independent mirtrons called simtrons which neither utilize DGCR8, Dicer, Exportin-5, or Argonaute 2 (Ago2) in their biogenesis (Havens et al., 2012).

**Figure 3:**
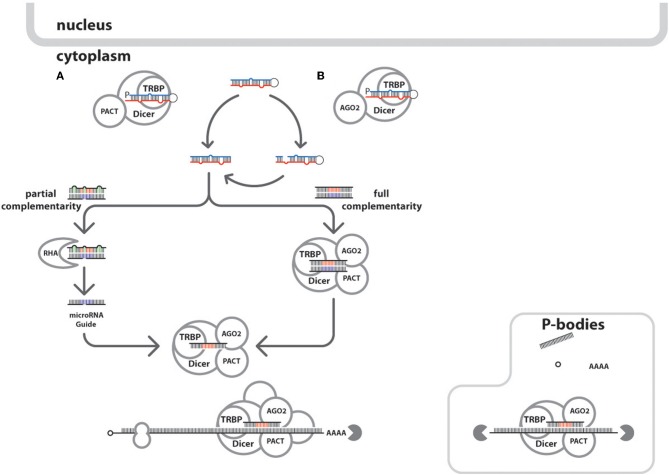
**Cytoplasmic events of the integrated cellular and HIV-1 microRNA biogenic pathways.** Mature microRNAs are processed in the host cytoplasm after the pre-microRNAs are shuttled from the nucleus. There are two ways the processing can happen: **(A)** by the Dicer/TRBPorTARBP or TARBP/PACT leading to the generation of microRNA duplexes, or **(B)** via the Ago proteins-assisted generation of pre-cursor microRNA which in the end generate the microRNA duplexes via Dicer action. The circular arrows show the formation microRNA duplex produced after the circuitous cleavage reactions of either Dicer, Ago2 proteins. Also shown in the microRNAs duplexes are the representative seed sites in **red** and blue and **green** indicates mismatches and bulges. Depending on the degree of complementation of the guide strands in the microRNA:RISC assembly to its target mRNA, would either cause mRNA degradation or translational repression. Furthermore, microRNA duplexes undergoing asymmetric unwinding would be assembled into the RISC loading complex after the duplex unwinding by RNA helicase A (RHA, gray crescent), through a bypass mechanism. Once the microRNA guide is loaded into the RISC associated proteins (Dicer; TRBP; Ago2; PACT; GW182, not shown) forms the activated microRNA:RISC to seek and bind targets within mRNA transcript. The mRNA transcripts are shown with other components namely: methyl cap (in hollow small circle), ribosome (hollow figure eight), and poly-A tail (AAAA). In HIV-1, it was demonstrated that P-bodies modulate microRNA processing mechanisms (Nathans et al., 2009). The solid gray pac-man indicates mRNA degradation events.

Then, the pre-microRNA associates with Ran/GTPase (Bohnsack et al., 2004; Okada et al., 2009) and exportins for cytoplasmic translocation from nucleus (Bohnsack et al., 2004). In the cytoplasm (Figure 3), catalytic hydrolysis of Ran/GTPase allows the dissociation of pre-microRNA and transporter proteins (Kim, 2004). Another enzyme called Dicer, splices the pre-microRNA into the mature ~22 nt microRNA capable of mRNA duplexing (Carmell and Hannon, 2004; Cullen, 2004; Hammond, 2005; Harvey et al., 2008; Flores-Jasso et al., 2009). A complement strand from the mature double stranded microRNA is integrated into the RNA-induced silencing complex (RISC) which would be attached to the target transcript to elicit the regulatory processes (Kawamata and Tomari, 2010).

The gene regulatory effects caused by the microRNA and mRNA target interaction dictated by highly stringent base-complementation of the binding sites have been demonstrated extensively (Long et al., 2008; Brodersen and Voinnet, 2009; Ajay et al., 2010). Perfect complementation of the microRNA and mRNA causes endonucleolytic cleavage-induced gene silencing while non-perfect complementation initiate translational inhibition of proteins (Jackson and Standart, 2007; Seitz, 2009; Pan et al., 2010). Other factors shown to enhance these regulatory interactions include the presence of several binding sites within the target mRNA and CTG repeats extension (Hon and Zhang, 2007), deadenylation and decapping-mediated conformational changes within the microRNA-RISC complex (Lin et al., 2005; Eulalio et al., 2007), the A-U bias (Frank et al., 2010) and single nucleotide polymorphisms (SNPs) within the seed regions (Landi et al., 2011).

Since, the viral genomic elements intersperse within the host genome during invasion, it is possible that these viral genomic fragments are processed into microRNAs by of the host microRNA machinery (Figures 2, 3). As consequence, these viral sequences will follow several pathways of microRNA biogenesis similar to its host. As example are the observed non-significant differences among microRNA profiles in normalized Dicer and Drosha expressions in HIV-1 infected CD4+ cells for both *in vitro* and *in vivo* studies (Bignami et al., 2012). In contrast, it was confirmed that microRNA expression among monocytes could happen in the absence of the Dicer enzyme, thus, implying an alternative mode of viral microRNA production (Coley et al., 2010). Another possible consequence of repeated integration and recombination of the HIV-1 into the host genome is the generation of orthologous microRNAs. This is the case of hiv1-miR-N367 and hsa-miR-192 with identical seed sequences shown to down regulate similar functional targets in a dual fluorescent reporter study, thus, making them functional orthologs (You et al., 2012).

## Alterations in the cellular microRNA pathways during HIV-1 infection

HIV-1 infection of host cells modifies the global RNA interference machinery which in effect changes the microRNA-regulated pathways via several bio-molecular interactions (Sanghvi and Steel, 2011b). Experiments confirmed the RNA silencing suppressor (RSS) activity of HIV-1 transactivator of transcription (Tat) (Bivalkar-Mehla et al., 2011). RSS is defined as a molecule encoded by a virus which can counter the effect of host cell microRNA-mediated antiviral defense pathways, or natural immunity (Houzet and Jeang, 2011). The HIV-1 Tat protein via association with the trans-activation response (TAR) element at the terminal 5′end of HIV-1 transcripts, promotes viral transcription by recruiting and increasing the processivity of RNA polymerase II (Hayes et al., 2011). The molecular complex creates a stabilizing effect on transcriptional elongation elicited by a cyclin-dependent kinase (CDK9), another subunit of the positive transcription elongation factor b (P-TEFb) together with Cyclin T1 (CCNT1), which functions to phosphorylate the C-terminus of RNA polymerase II (Sanghvi and Steel, 2011a). Also the binding between Tat protein to the TAR element blocks the TAR element interaction with the Dicer protein thus influencing cellular silencing mechanism (Bennasser and Jeang, 2006; Qian et al., 2009). Further characterization of Tat protein exhibited two most essential prerequisites for RSS activity, namely by harboring dsRNA binding domain (RKKRRQRR) and GW/WG motif essential in sequestering Ago proteins thus preventing RISC formation (Qian et al., 2009; Bivalkar-Mehla et al., 2011; Houzet and Jeang, 2011). In another comparative gene expression profile analysis between HIV-1 infected and non-infected macrophages, it was showed that HIV-1-encoded Vpr (Viral Protein R) protein similarly suppresses Dicer function (Coley et al., 2010). Table 1 includes the RNAi pathway related gene products that are targeted by HIV-1 microRNAs. With the inherent small size of microRNAs, bi-target co-regulation is a prospective occurrence when a microRNA seed sequence would complement both viral and cellular mRNAs simultaneously due to sequence similarities. Though, the mechanisms of these interactivities are not thoroughly explainable at the moment, computational analyses suggest the probabilities of their existence, in particular during viral infection (Veksler-Lublinsky et al., 2010).

**Table 1:** **List of published HIV-1 microRNAs and their target HIV-1 and cellular gene products**.

**HIV-1 microRNA Name (A)**	**Mature sequence variants (B)**	**Gene product (mRNA) targets (C)**	**Function of mRNA targets (D)**	**References**
hiv1-miR-TAR-5p	**4-UCUCUCUGGUUAGACCAGAUCUGA-27 (Ra)**	**LTR *d***	Viral gene expression	Klase et al., 2007, 2009; Ouellet et al., 2008; Schopman et al., 2012
	**UGGGUCUCUGGUUAGACCAG (Bp)**	**TAR *d***	Regulation/anti-apoptosis	
	**GGUCUCUGGUUAGACCA (Nb)**	**RITS *d***	Viral co-factor	
	**GGGUCUCUCUGGUUAGACCA (Nb)**	ERCC1(apo) ***d***	Cell apoptotic factor	
		IER3 (apo) ***d***	Cell apoptotic factor	
	**CUCUGGCUAACUACUAGGGAACCC (Ns)**			
	**UCUGGCUAACUACUAGGGAA (Ns)**			
	**UCUGGCUAACUACUAGGGAACCCA (Ns)**			
	**CUGGCUAACUACUAGGGAA (Ns)**			
	**UGGCUAACUACUAGGGAA (Ns)**			
	**UGGCUAACUACUAGGGAACCCAC (Ns)**			
	**UGGCUAACUACUAGGGAACCCACU (Ns)**			
	**UGGCUAACUACUAGGGAACCCACUG (Ns)**			
	**GGCUAACUACUAGGGAACCCACUG (Ns)**			
	**CUAACUACUAGGGAACCCACUGC (Ns)**			
hiv1-miR-TAR-3p	**38-UCUCUGGCUAACUAGGGAACCCA-60 (Ra)**	**TAR *d***	Viral gene expression	Klase et al., 2007; Schopman et al., 2012
	**CUAACUAGGGAACCCAC (Nb)**	**RITS *d***	Regulation/anti-apoptosis	
	**GCUAACUAGGGAACCCAC (Nb)**			
	**GCUAACUAGGGAACCCACUG (Nb)**			
hiv1-miR-H1	**2-CCAGGG-AGGCGUGCCUGGGC-21 (Nb)**	AATF ***d***	Adaptive immunity	Kaul et al., 2009; Lamers et al., 2010
	**CCAGGG-AGGCGUGgCaUGGGC (Mc)**	**BCL2 *d***	Activated cell	
	**CCAGGG-AGGCGUGgCCUGGGC (Mc)**	MYC ***d***	Proliferation regulator	
	**CCAGGG-AGGCGUGgCCUGGGC (Mc)**	PAWR ***d***; **DICER *d***	Pro-viral latency promoter	
	**CCAuGGgAGGCGcGgCCUGGGC (Mc)**			
	**CCAGGG-AGGCGUGgCCgGGGu (Mc)**	hsa-miR-194 ***d***	MicroRNA processing	
	**CCAGGGgAGGCGUGaCCUGGGC (Mc)**		Pre-microRNA	
			Processing/microRNA binding	
hiv1-miR-N367	**40-ACUGACCUUUGGAUGGUGCUUCAA-62 (Nb, Mc)**	**Nef *d***	Transcription factor and regulator	Omoto and Fujii, 2005

Notes: **(A)** The official names of microRNAs as published in mirbase.org. ***(B)*** The sequences of mature HIV-1 microRNA variants as determined by several methods including: Bp, bioinformatic prediction; Ra, RNase protection assays; Nb, Northern blotting; Ns, next generation sequencing; Mc, molecular cloning. Please note that sequences are not aligned accordingly; lowercase letters indicate polymorphic sites when available. The numbers before and after the nucleotide sequences refer to the relative genomic position/s in the pre-microRNA sequences if made available by authors in literature. ***(C)*** The mRNA targets of the HIV-1 microRNAs immediately followed by italicized letters correspond to the type of regulation, where: u = up-regulation, d = down-regulation when described in literature. In addition, if targets are HIV-1 mRNA genes or mRNA transcripts they are typed in ***boldface***, RNAi pathway-related gene products typed in **BLUE**; and literature-based standard HIV-1 linked cellular gene products are typed in **RED**. ***(D)*** The reported functional attributes of mRNA targets by HIV-1 microRNA among studies.

## Alterations in cellular microRNA expression during HIV-1 infection

HIV-1 infection induced changes in cellular microRNA expressions result from combinatorial molecular interactions among proteins, transcripts, and genomes. These manifest as circuitous microRNA attenuation of the different cellular host metabolic processes. At this point, the current knowledge of the exact mechanisms on how HIV-1 infection remains to be understood fully. The current data available are mostly derived from microarray data comparing non-infected and HIV-1 infected cell lines. Over expression analyses of microRNAs among various cell lines simulated with HIV-1 infection are usually used to validate these differences in an attempt to explain the possible regulatory mechanisms behind the miroRNA interactions. Examples include the (Houzet et al., 2008) investigation which reported 59 simultaneously down-regulated cellular microRNAs of HIV-1 infected individuals. Prior studies indicated that HIV-1 infection can down-regulate as much as 43% of the 312 microRNA gene arrays (Yeung et al., 2005). In addition, unique and variable global modifications in microRNA expressions were exhibited by different host cell types and lines in reaction to HIV-1 infection (Yeung et al., 2005; Bennasser et al., 2006; Noorbakhsh et al., 2010; Gupta et al., 2011). A most recent example is the notable differences among the 21 microRNA profiles between the elite long-term non-progressors where viral replication is continuously suppressed against multiple uninfected individuals from 377 microRNAs changes in HIV-1 infected CD4+ lymphocytes (Bignami et al., 2012). Determining the alterations in cellular microRNA expression patterns among various types of HIV-1 infected cell lines may account factors such as productive infection and constant exposure to HIV-1 that drive these changes. Also modifications in microRNA profiles may be attributed to temporal variability of immune responses during the course of HIV-1 infection. When fully elucidated, these patterned variations of microRNA expressions may reflect vital information in HIV-1 disease diagnostics and progression. Currently, microarray data has been the greatest source of these analyses of microRNA expression pattern changes, aided by complex algorithms to detect actual variation.

An in depth analyses of these global changes confirm the existence of clustered microRNA expression signatures in HIV-1 infected cells. For example, downregulation of polycistronic microRNA hsa-miR-17/92 consistently suppressed viral production as observed among various HIV-1 infected cells (Triboulet et al., 2007). While, hsa-miR-27b, hsa-miR-29b, hsa-miR-150, and hsa-miR-223 were identified as significantly down-regulated upon CD4(+) T cell activation (Chiang et al., 2012). In contrast, hsa-miR-28, hsa-miR-125b, hsa-miR-150, hsa-miR-223, and hsa-miR-382, which were enriched in resting CD4+ T cells against the activated CD4+ T cells (Huang et al., 2007). Moreover, the T-cell-specific microRNAs, namely hsa-miR-150, hsa-miR-191, hsa-miR-223, hsa-miR-16, and hsa-miR-146b, showed variable expression patterns at various stages of HIV-1 infection. Recently, gene expression profile analyses of cellular microRNAs in HIV-1 infected CD4+ T cells demonstrated the down-regulation of hsa-miR-21, hsa-miR-26a, hsa-miR-155, hsa-miR-29a, hsa-miR-29b, and hsa-miR-29c, contrary to the observed upregulation of hsa-miR-223 (Sun et al., 2012). The identification of microRNA families may hold significance in correlating the targets as orthologous modules as previously mentioned.

## HIV-1 encoded microRNAs and their interactions

The low number of verified HIV-1 encoded microRNAs (Table 1) in the miRBase (2012) confirm the difficulty of their identification thus making them among the least characterized of RNA virus-generated microRNAs (Grundhoff and Sullivan, 2011). This scarcity may be due to their inherent low number because of the small genome size or low levels of expression currently undetectable by conventional biochemical techniques thus may require enrichment processes to be detected (Althaus et al., 2012). Previously, Lin and Cullen (2007) estimated that retroviral microRNAs comprise only 0.5% of the total microRNAs detectable in HIV-1 infected cells. In addition, the limited access of viruses to nuclear microRNA processing machinery and the natural destabilization effects of microRNA processing may also limit their biogenesis (Grundhoff and Sullivan, 2011). Also, several reports confirmed the endonucleolytic effects of Dicer or Drosha against viral RNA genomes thus reducing viral mRNA production (Ouellet and Provost, 2010).

However, the advent of highly sensitive technologies like next generation sequencing and RNAse protection assays (RPA), as well as improved computational prediction may contribute to the discovery of new HIV-1 microRNA species. Recent pyrosequencing results estimated at least 40% or 125 of the candidates as putative HIV-1 microRNAs originating from the TAR, RRE and *nef* region, and major components of non-coding RNAs in HIV-1 infected cells (Yeung et al., 2009). The deep sequencing report of (Schopman et al., 2012) further supports these observations, as HIV-1 microRNAs are suggested to arise from structured regions of the genome which facilitate Drosha and Dicer mediated RNA processing. Hence, with the increased possibilities that many putative HIV-1 microRNAs identified by these breakthrough procedures, they require further investigations on their isolation and functional characterization.

In general, the target interactions of HIV-1 microRNAs with its mRNA seem to function as viral genome regulators (Table 1). However, current experiments open this into a subject of debate and further investigation. Although, functional studies suggest auxiliary functions of HIV-1 microRNAs which target host cellular transcripts mainly for immune evasion (Boss and Renne, 2011). These observations are explained further in succeeding discussions below.

### HIV-1 TAR microRNA

The 50 nt HIV-1 TAR element within the 5′ region of the viral RNA serves as the progenitor of hiv1-miR-TAR via asymmetrical processing of the transcript (Ouellet et al., 2008). Experiments confirmed hiv1-miR TAR to target host cell microRNA-related proteins, namely, Dicer, trans-activation responsive RNA binding protein (TARBP2, TRBP), and the RNA induced transcriptional silencing (RITS) complex. Recent functional studies showed that HIV-1 TAR microRNA down-regulates the DNA excision repair (ERCC1) and radiation-inducible immediate-early gene IEX-1 proteins (IER3) thus exerting its anti-apoptotic effect in infected cells (Klase et al., 2009).

Computer simulation studies established the HIV-1 TAR element as a potential microRNA rich region because of the following evidences (Narayanan et al., 2011): (a) the hairpin formation of the TAR element concurs with Dicer substrate specifications, allowing the complement fit to five distinct Dicer element, (b) TAR binds to important microRNA proteins, Dicer, and TARBP2, thus singling out its essential role in the microRNA-mediated gene regulatory processes. Moreover, the TARBP2 association with Dicer is necessary for efficient loading of microRNAs into the RISC, the consequential loss of TARBP2 function culminates in the loss of RNA silencing ability (Sanghvi and Steel, 2011a). The TARBP2 sequestration is known to restrict the availability of Dicer enzyme leading to modification of microRNA processing (Haase et al., 2005). Furthermore, TARBP2 and TAR element association suppresses interferon (IFN)-induced protein kinase R function (Gatignol et al., 2005)

Cloning studies of TAR-related microRNAs demonstrated a greater abundance of the 3′ mature sequence over the 5′ mature sequences involved in microRNA-derived silencing (Lamers et al., 2010). These observations collectively suggest that, in infected cells, hiv1-miR-TAR-3p is superior to the hiv1-miR-TAR-5p in suppressing gene expression, supporting speculations that there are preferential releases of these microRNA species. This also corroborates to the evident accumulation of the 3′ HIV-1 TAR RNAs *in vivo* (Ouellet et al., 2008).

### HIV-1 H1 microRNA

The 81bp stem loop of HIV-1 transcript formed in the 3′-U3 (LTR) region known as the binding sites of the two nuclear factor kappa-light-chain-enhancer of activated B cells (NF-kB) is the origin of hiv1-miR-H1 (MI0006106). It was shown to degrade apoptosis antagonizing factor (AATF) which decreases cell viability and reduced expression of cellular factors, Bcl-2, c-myc, Par-4 as well as the microRNA Dicer protein (Kaul, 2007; Kaul et al., 2009). The report also indicated hiv1-miR-H1 interaction with hsa-mir-149, affecting the latter's target HIV-1 encoded Vpr protein (Kaul et al., 2009). It is assumed that hiv1-miR-H1 and hiv1-miR-TAR are antagonistic to one another as they have contrasted activities against apoptotic elements. Further functional studies demonstrated deletion-driven evolution patterns in hiv1-miR-H1 among various AIDS patients. In addition, causal association was suggested between the appearance of a less stable hiv1-miR-H1 variant and induction of AIDS-related lymphoma (Lamers et al., 2010).

### HIV-1 Nef microRNA

Nef protein has been shown to downregulate cell surface CD4 and MHC class I molecules through the clathrin-mediated endocytic pathway (Lubben et al., 2007; Schaefer et al., 2008). It is also involved in cellular signal transduction pathway through interaction with non-receptor type Tyr kinase molecules such as, Fyn and Lyn. Since, Nef functions in favor of HIV-1 replication and it is relatively conserved among various HIV-1 variants, miRNA-mediated control of Nef could have a great effect on the viral life cycle and its pathogenesis (Arien and Verhasselt, 2008; Foster and Garcia, 2008; Malim and Emerman, 2008). Although, HIV-1 3′ LTR is partially overlapping with *nef* microRNA (hiv1-miR-N367), as proposed previously (Omoto and Fujii, 2005, 2006), it remained controversial due to its non-duplicability. These reports may support a hypothesis of hyper-evolution of HIV-1 genome as a consequence of peptide-based immunity and RNA interference mechanisms (Narayanan et al., 2011).

### HIV-1 antisense microRNAs

Recent reports have indicated the ability of HIV-1 utilizing antisense transcripts in infected cells leading to discoveries of new viral microRNAs. In the RACE analyses of HIV-1 infected 293T and Jurkat cells, it was shown that cryptic transcription initiation sites in the 5′ border of the 3′ LTR and a new poly A signal within this LTR were present; also indicated was the possible role of the Tat protein as the modulator of transcription of this antisense RNA (Landry et al., 2007). In another study, an antisense peptide open reading frame (ORF) called “asp” coding for a hydrophobic protein was derived from Jurkat cells infected with HIV-1 although its origin, generation or the function is not yet clarified (Clerc et al., 2011). As the existence of antisense HIV-1 microRNAs remains to be proven, this concept opens a possibility where long antisense transcripts can complement with the sense transcripts within the viral genome. These sites in double stranded configuration can provide biogenic zones of Dicer-mediated microRNAs (Houzet and Jeang, 2011).

## Cellular microRNAs involved in HIV-1 infection

HIV-1 infection triggers multi-modal cascades of host cell microRNA targeting interactions that either activate or inhibit viral invasion and replication as shown in Figure 1. These microRNA targeting scenarios are likely to occur on at least two fronts. First, the cellular microRNA might directly target the HIV-1 genome, either in sense or antisense orientation, to suppress the production of viral proteins. An outstanding example is hsa-miR-29 which targets the HIV-1 nef transcript (Hariharan et al., 2005; Ahluwalia et al., 2008). Since, the HIV-1 nef gene is located at the proviral DNA 3′ portion, cellular microRNA targeting of this region would have serious implications in the viral life cycle. The group of (Nathans et al., 2009) proved that ectopic expression of hsa-miR-29 can repress production of *nef* protein resulting to suppressed viral replication and infectivity. The study also reported that hsa-mir-29a/ HIV-1 interactions enhance viral mRNA associations with RISC and P-body structures, thus suggesting prospective roles of P-bodies to viral latency regulation. In another study, a set of microRNAs namely, hsa-mir-28, hsa-miR-125b, has-miR-150, hsa-miR-223, and hsa-miR-382 were shown to bind in the 3′ position of HIV-1 transcripts which triggers viral latency (Huang et al., 2007). Recently, Sun et al identified another set of cellular microRNA, namely hsa-miR-15a, hsa-miR-15b, hsa-miR-16, hsa-miR-24, hsa-miR-29a, hsa-miR-29b, hsa-miR-150, and hsa-miR-223 that are directly targeting HIV-1 3′-UTR, and exhibiting weak but significant inhibitory effects on HIV-1 replication (Sun et al., 2012). In a review by Sun and Rossi, using the PITA software, 256 seed-match sites were identified to complement Nef-3′ LTR sequence (Sun and Rossi, 2011).

In the second scenario, the host cell as triggered by HIV-1 infection would initiate cellular microRNA production to attenuate cellular factors involved in antiviral responses against HIV-1. Table 2 summarizes the list of cellular microRNAs with their validated host cellular protein targets and their corresponding cellular functions. As likely initial repercussions, these microRNAs may target the genes involved in immune responses for innate and adaptive immunity (Kulpa and Collins, 2011). This bipartite defense system initially triggers natural killer (NK) cell activities as elicited by partial detection of HIV-1 components. In a later reaction, adaptive immunity is induced through production of antigen-specific antibodies by B-cells and eliciting cell-mediated immunity through antigen-specific cytotoxic T lymphocytes, of which microRNAs were found to target various cellular receptors (Cobos-Jimenez et al., 2011). As examples are cellular microRNA interactions with chemokines (Zhou et al., 2010).

**Table 2:** **List of published cellular microRNAs and their target HIV-1 and cellular gene products**.

**Cellular microRNA Name (A)**	**Gene product (mRNA) targets (B)**	**Function (C)**	**References**
hsa-let-7/ g^*^	**DICER**	Pre-microRNA processing/microRNA binding	Faller and Guo, 2008; Faller et al., 2010; Desjardins et al., 2012; Swaminathan et al., 2012
	**LIN28**	Pre-microRNA processing regulation; repress maturation of hsa-let-7 family; blocks Drosha and Dicer processing of pri-/pre hsa-let-7 family via interaction with terminal loop; blocks Dicer processing of pre- hsa-miR-128	
	**IL-10**	Inflammatory response	
hsa-miR-17^*^/17-3p	**EP300**/CBP associated factor (PCAF) ***u***	Transcription factor and regulator/control of viral replication	Triboulet et al., 2007; Hayes et al., 2011
hsa-miR-17/17-5p	KAT8	HIV-1 Tat interactive protein	
	HA Tat co-factor	HIV-1 Tat interactive protein	
hsa-miR-92a-1^*^	HA Tat cofactor	HIV-1 Tat interactive protein	Sun et al., 2012
hsa-miR-125b-5p	**Nef-3′ UTR LTR**	Viral replication and promotion of viral latency in T-cells	Huang et al., 2007; Witwer et al., 2012
hsa-miR-125b-1^*^/125b-2^*^			
hsa-miR-125a-5p			
hsa-miR-125a-3p			
hsa-miR-128	SNAP25	Cellular receptor	Eletto et al., 2008
hsa-miR-146	**CCL8/MCP-2**	Innate immune response factor	Rom et al., 2010
hsa-miR-149	**Vpr**	Regulation of nuclear import of HIV-1 pre-integration complex; viral replication and cellular immune suppression	Kaul et al., 2009
hsa-miR-150/150^*^	**3′ end of HIV-1 RNA**	Viral replication and promotion of viral latency in T-cells	Huang et al., 2007; Witwer et al., 2012
	**APOBEC3G/3F *d***	Cellular co-factor; relieves microRNA repression mechanisms	
	**CCR5**	HIV-1 receptor and natural ligand	
	**CD4 *d***	HIV-1 receptor and natural ligand	
	**CCNT1**	Repression of HIV-1 tat co-factor for transcriptional *trans*-activation	
hsa-miR-155	Target not specified	Function not specified	Sun et al., 2012
hsa-miR-198	**CCNT1** (P-TEFb) ***d***	Repression of HIV-1 Tat co-factor for transcriptional *trans*-activation	Sung and Rice, 2009
hsa-miR-20a	PCAF ***u***	Co-factor of Tat *trans*-activation.	Hayes et al., 2011
	KAT8	Cellular transcription activator	
	MCL1	Cellular anti-apoptotic factor	
	DNMT3A/B	Cellular transcriptional regulator	
	TCL1A	Interacts with IKB	
	PIC3R1	PI3 kinase subunit	
	**CDC42**	HIV-1 receptor and natural ligand	
hsa-miR-21	Target not specified	Function not specified	Sun et al., 2012
hsa-miR-27a^*^/27a	**CCNT1**	Transcription factor and regulator; repression of HIV-1	Chiang et al., 2012
hsa-miR-27b^*^/27b		Tat co-factor for transcriptional *trans*-activation	
**hsa-miR-28-5p/28-3p**	**3′ end of HIV-1 RNA**	Viral replication and promotion of viral latency in T-cells	Huang et al., 2007; Swaminathan et al., 2009
	**CCR5**	HIV-1 receptor and natural ligand	
	**CD4 *d***	HIV-1 receptor and natural ligand	
	**APOBEC3G/3F *d***	Cellular co-factor	
**hsa-miR-29a**/29a^*^	**Nef protein coding mRNA**	Viral replication and latency	Ahluwalia et al., 2008; Nathans et al., 2009; Chiang et al., 2012; Sun et al., 2012; Witwer et al., 2012
hsa-miR-29-b1^*^/**29b^1d^**/29-b2^*^	3′-**UTR** (420)	Viral replication and latency	
hsa-miR-29c^*^/**29c**	**RISC**, P bodies	Mature microRNA assembly/carrier	
	MCL-1		
	DNMT 3A/B	Cellular anti-apoptotic factor	
	TCL1a, p85a	Cellular transcriptional regulator Interacts with IKB	
	CDC42	PI3 kinase subunit	
	**CCNT1**	HIV-1 receptor and natural ligand	
		Transcription factor and regulator; repression of HIV-1	
		Tat co-factor for transcriptional *trans*-activation	
hsa-miR-217	SIRT1	Cellular stress response regulator	Zhang et al., 2012
hsa-miR-223^*^/**223**	**3′ end of HIV-1 RNA**	Viral replication and promotion of viral latency in T-cells	Huang et al., 2007; Swaminathan et al., 2009; Chiang et al., 2012; Sun et al., 2012
	**APOBEC3G/3F *d***	Cellular co-factor	Chiang et al., 2012
	P3 *d*	Cellular co-factor	
	LIF *d*	Cellular co-factor	
	RobB *d*	Cellular co-factor	
	**CCNT1**	Transcription factor and regulator	
hsa-miR-31/31^*^	Target not specified	Function not specified	Witwer et al., 2012
hsa-miR-34a	**CREBBP**	Transcription factor and regulator	Chiang et al., 2012
hsa-miR-382	**3′ end of HIV-1 RNA**	Viral replication and promotion of viral latency in T-cells	Huang et al., 2007

Notes: ***(A)*** The official names of microRNAs as published in mirbase.org. The microRNAs in ***boldface*** are the dominant targeting species when reported in literature. ***(B)*** The mRNA targets of the HIV-1 microRNAs are immediately followed by italicized letters which correspond to the type of regulation, where: u = up-regulation, d = down-regulation when described in literature. In addition, if targets are HIV-1 mRNA genes or mRNA transcripts they are typed in ***boldface***, RNAi pathway-related gene products typed in **BLUE**; and literature-based standard HIV-1 linked cellular gene products are typed in **RED**. ***(C)*** The reported functional attributes of mRNA targets by HIV-1 microRNA among studies.

Transcriptional control is vital to the HIV-1 proliferation, thus determining microRNA interactions among host transcription factors and regulators is a necessity (Victoriano and Okamoto, 2012). Among examples are reporter assays suggesting hsa-miR-223 bi-functional effects in HIV-1 replication are targets were varied in two different cell lines namely, Sp3 and LIF in NB4 cells, while RhoB and NF-1A in HEK293 cells (Sun et al., 2012). Next is the hsa-miR-29 family which also targets cellular proteins Mcl-1, DNMT 3A/B, Tcl1, p85, and CDC42, further establishes its diverse roles in HIV-1 latency (Sun et al., 2012; Witwer et al., 2012). Another is hsa-miR-198 targeting CCNT1 (Kaul et al., 2009), a key component in the Tat-mediated transcription of the virus, when suppressed can impair HIV-1 replication (Imai et al., 2009).

Cellular microRNAs linked to chromatin regulation show proof that microRNAs are critical elements of epigenetic control in HIV-1 infection (Obbard et al., 2009; Easley et al., 2010). Recent evidences support that chromatin modification may explain mechanisms of HIV-1 transcription and thus the maintenance of latency. Some microRNA species are considered involved in gene silencing by modulating methylation and deactylation of histone proteins (Triboulet et al., 2007).

The above mentioned functional gene product clusters are just few focal points of cellular microRNA interactivities related to HIV-1 infection. It is expected that as more interactions are validated, the complex nature of cellular microRNA regulation linked to HIV-1 infection and host response would be further characterized. However, the scope of cellular microRNA interactions may involve other non-listed prospective gene targets which may also influence HIV-1 infection.

## Beyond crosstalks among cellular and HIV-1 microRNA machineries

Preceding discussions on microRNA interactions in host-HIV-1 infection further confirm their inherent complexity. It perfectly illustrates the constant attenuation of gene regulatory networks to maintain homeostasis in the HIV-1 infected cells. However, as HIV-1 remains an incurable disease among humans, it is implied that it can successfully compromise host immune and defense reactions wherein microRNA regulation might play pivotal roles. Thus, future studies must focus on how to reprogram microRNAs to favorably initiate the cellular anti-HIV-1 defense response. To realize such goal, it becomes necessary to organize succeeding investigations as follows: First is to globally account cellular and viral microRNA interrelationships affecting biomolecular pathways in HIV-1 infection. This allows the possibility of unlocking the combination of molecular switches that would allow the host cell successfully defend itself against HIV-1. Second is to determine the simultaneous targets of viral and cellular microRNAs. These bi-targets may reveal signatures of gene families or microRNA clusters characterizing HIV-1 infection patterns. Third is to capture temporal changes among microRNA expression patterns during HIV-1 disease progression. In assessing the current amount of information on hand, there remains much work to be done in unlocking the ultimate roles of microRNAs in HIV-1 pathogencity.

### Conflict of interest statement

The authors declare that the research was conducted in the absence of any commercial or financial relationships that could be construed as a potential conflict of interest.
